# Differential Patterns and Determinants of Cardiac Autonomic Nerve Dysfunction during Endotoxemia and Oral Fat Load in Humans

**DOI:** 10.1371/journal.pone.0124242

**Published:** 2015-04-20

**Authors:** Dan Ziegler, Alexander Strom, Klaus Strassburger, Bettina Nowotny, Lejla Zahiragic, Peter J. Nowotny, Maren Carstensen-Kirberg, Christian Herder, Julia Szendroedi, Michael Roden

**Affiliations:** 1 Institute for Clinical Diabetology, German Diabetes Center at Heinrich Heine University, Leibniz Center for Diabetes Research, Düsseldorf, Germany; 2 Department of Endocrinology and Diabetology, University Hospital, Düsseldorf, Germany; 3 German Center for Diabetes Research (DZD), Partner Düsseldorf, Düsseldorf, Germany; 4 Institute of Biometrics and Epidemiology, German Diabetes Center at Heinrich Heine University, Leibniz Center for Diabetes Research, Düsseldorf, Germany; University of Milan, ITALY

## Abstract

The autonomic nervous system (ANS) plays an important role in regulating the metabolic homeostasis and controlling immune function. ANS alterations can be detected by reduced heart rate variability (HRV) in conditions like diabetes and sepsis. We determined the effects of experimental conditions mimicking inflammation and hyperlipidemia on HRV and heart rate (HR) in relation to the immune, metabolic, and hormonal responses resulting from these interventions. Sixteen lean healthy subjects received intravenous (i.v.) low-dose endotoxin (lipopolysaccharide [LPS]), i.v. fat, oral fat, and i.v. glycerol (control) for 6 hours, during which immune, metabolic, hormonal, and five HRV parameters (pNN50, RMSSD, low-frequency (LF) and high-frequency (HF) power, and LF/HF ratio) were monitored and energy metabolism and insulin sensitivity (M-value) were assessed. LPS infusion induced an increase (AUC) in HR and LF/HF ratio and decline in pNN50 and RMSSD, while oral fat resulted in elevated HR and a transient (hours 1-2) decrease in pNN50, RMSSD, and HF power. During LPS infusion, ΔIL-1ra levels and ΔIL-1ra and ΔIL-1ß gene expression correlated positively with ΔLF/HF ratio and inversely with ΔRMSSD. During oral fat intake, ΔGLP-1 tended to correlate positively with ΔHR and inversely with ΔpNN50 and ΔRMSSD. Following LPS infusion, lipid oxidation correlated positively with HR and inversely with pNN50 and RMSSD, whereas HRV was not related to M-value. In conclusion, suppression of vagal tone and sympathetic predominance during endotoxemia are linked to anti-inflammatory processes and lipid oxidation but not to insulin resistance, while weaker HRV changes in relation to the GLP-1 response are noted during oral fat load.

**Trial Registration:**

ClinicalTrials.gov NCT01054989

## Introduction

The metabolic homeostasis is regulated by the vagus nerve via controlling heart rate, gastrointestinal motility and secretion, pancreatic endocrine and exocrine secretion, and endogenous glucose production [1,2]. The parasympathetic nervous system (PNS) also controls innate immune responses and inflammation during pathogen invasion and tissue injury. The physiological mechanism through which the PNS regulates immune function and inhibits excessive proinflammatory cytokine production has been termed the “inflammatory reflex” [1–3]. Vagus nerve signaling plays a paramount role in the regulation of feeding behavior and metabolic homeostasis aimed at preserving energy balance and preventing fluctuations in body weight and metabolism [1]. The efferent vagus nerve signaling pathway termed the “cholinergic antiinflammatory pathway” has been corroborated in an animal model of endotoxemia and shock [3–5]. Cholinergic agonists inhibit cytokine synthesis and protect against cytokine-mediated diseases [5], and the myocardial inflammatory response and impairment during experimental endotoxemia are aggravated following vagotomy [6]. The sympathetic nervous system (SNS) plays a dual role in the regulation of inflammation by mediating both pro- and anti-inflammatory activities [4]. Both divisions of the autonomic nervous system (ANS) are activated by immunogenic stimuli and both contribute to modulation of inflammation. The cholinergic anti-inflammatory pathway and the SNS also act synergistically to control inflammation [4].

Analysis of heart rate variability (HRV), which measures the fluctuations in autonomic inputs to the heart [7], is considered a prognostic marker in various conditions including diabetes [8], cardiovascular disease [7], and multiple organ dysfunction syndrome as a consequence of sepsis [9]. Both branches of the ANS are affected by the septic response and, vice versa, modulate the immune response during sepsis [10,11]. Critically ill patients with evidence of systemic inflammation due to sepsis showed reduced HRV which was related to disease severity [10,12] and increased systemic interleukin 6 (IL-6) levels [13]. Experimental endotoxemia induced by lipopolysaccharide (LPS) administration mediating systemic inflammation as a model of the initial septic response leads to a considerable decrease in HRV indices [14–18] and baroreflex dysfunction with uncoupling of heart rate modulation to blood pressure changes [19]. The most frequently measured cytokines in studies of low-dose endotoxemia are the pro-inflammatory cytokines IL-6 and TNFα and the anti-inflammatory IL-1 receptor antagonist (IL-1ra) [20]. IL-1ra is, like IL-6 and TNFα, upregulated in response to the pro-inflammatory immune activation triggered by LPS but is showing later peak concentrations in the circulation than IL-6 and TNFα. However, these three inflammatory parameters are the main components of the resolution of the acute immune response to LPS. Currently, it is unclear whether the depression of cardiac autonomic tone is linked to the pro- and anti-inflammatory response, since the extent of the inflammatory response did not correlate with the magnitude of HRV reduction [16,17] or heart rate [19], despite pronounced cardiac ANS changes after LPS administration. Since experimental data indicate that higher basal vagal activity leads to a less pronounced proinflammatory response via the cholinergic anti-inflammatory pathway [3], the positive correlation of baseline vagal activity with the maximal TNF-α response to LPS found in healthy humans appears contradictory [16]. Moreover, glucocorticoid-induced attenuation of the inflammatory response to endotoxin is not accompanied by diminished vagal or sympathetic tone [14], and in subjects who were administered LPS twice, inflammatory cytokines were markedly attenuated after the second LPS administration, whereas LPS-induced HRV alterations were similar [17]. On the other hand, epinephrine infusion prior to administration of endotoxin diminished the inflammatory response [15], and voluntary SNS activation resulted in epinephrine release and subsequent suppression of the innate immune response [18].

Spectral analysis of HRV indicates that efferent vagal activity is a major contributor to the high-frequency (HF) component, while the low-frequency (LF) component has been considered as a marker of sympathetic modulation or as being under both sympathetic and vagal influences [7]. Although the concept of sympathovagal balance remains a matter of ongoing discussion [21], a shift of cardiac ANS activity toward sympathetic predominance estimated by an increased LF/HF ratio has been linked to reduced glucose ultilization [22], increased free fatty acid (FFA) concentration [23], insulin resistance [24], and several components of the metabolic syndrome [25]. HF power was suppressed and the LF/HF ratio was increased during lipid infusion, suggesting that the latter may lead to SNS activation [23], but the effect of oral high-fat loading has not been studied. During the euglycemic—hyperinsulinemic clamp, an increase in the LF/HF ratio was found in lean subjects [26]. Likewise, higher fasting plasma concentrations of glucagon-like peptide 1 (GLP-1) were associated with reduced vagal activity and higher resting fat oxidation [27]. Low-dose GLP-1 administration leading to physiological postprandial plasma GLP-1 levels inhibited efferent vagal-cholinergic activity independent of intraduodenal lipid perfusion [28]. It has been suggested that insulin and GLP-1 share common effects that can contribute to vagal depression and sympathetic activation [29].

Against this complex background, we studied the effects of experimental conditions mimicking inflammation and hyperlipidemia on HRV and heart rate in relation to the immune, metabolic, and hormonal responses resulting from these interventions. We aimed to determine in lean healthy volunteers whether 1.) changes in cardiac autonomic tone following endotoxemia are linked to the pro- and anti-inflammatory response, 2.) lipid infusion and oral high-fat loading result in changes in HRV, and 3.) putative changes in autonomic tone are related to insulin sensitivity, substrate oxidation, and hormonal changes.

## Materials and Methods

### Subjects

All participants gave their written informed consent before inclusion into the Fat, Inflammation and Insulin Resistance (FIRE) study (ClinicalTrials.gov identifier number NCT01054989), which was performed according to the Declaration of Helsinki and approved by the local institutional ethics board of Heinrich Heine University, Düsseldorf, Germany. Inclusion criteria were age between 20 and 40 years and BMI between 20 and 25 kg/m^2^. Exclusion criteria were prediabetes, diabetes, menstrual irregularities, family history of diabetes, history of smoking and acute or chronic diseases including cancer, and medication affecting insulin sensitivity, lipid metabolism, or immune system. Screening included a standardized 75-g oral glucose tolerance test, routine laboratory tests, bioimpedance analysis to obtain lean body mass, and a questionnaire on habitual physical activity. Women were studied between days 5–9 of their menstrual cycle. All participants were assigned to 4 study days at least 4 weeks apart in random order and were instructed to maintain normal physical activity and a carbohydrate-rich diet for 3 days before all study days [30].

### Study design

The details of the study protocol have been reported elsewhere [30]. In brief, participants received each of four interventions: *1*) oral fat: 100 mL of soy bean oil (61% polyunsaturated, 23% monounsaturated, and 16% saturated fatty acids; Sojola; Vandemoortele Deutschland GmbH, Dresden, Germany) consumed within 10 min; *2*) LPS: 10-min infusion of LPS (0.5 ng/kg body weight; National Reference Endotoxin, *Escherichia coli O*:*113*; USP, Rockville, MD); *3*) intravenous fat (iv fat): infusion of Intralipid (1.5 mL/min; Fresenius Kabi GmbH, Bad Homburg, Germany) over 6 h; or *4*) control: 2.5% glycerol infusion dissolved in 0.9% saline (1.5 mL/min; Fresenius Kabi GmbH) for 6 h. Study participants were initially randomly assigned to the four intervention blocks (4 subjects per group) and thereafter allocated to the remaining interventions block by block. The time between the individual interventions was at least four weeks for each subject. Glycerol was chosen as a control, since infusion of lipid emulsion is associated with a rise in plasma glycerol concentration [23] and the primary goal of the study was to examine the initial events occurring during the onset of insulin resistance upon high systemic lipid load [30]. Saline would have been a more appropriate control for LPS and oral fat, but a previous study suggested that glycerol and saline solutions have similar effects on HRV measures [23].

Each intervention was followed by a hyperinsulinemic-euglycemic clamp as previously described [30]. Whole-body glucose disposal (M-value) was calculated from glucose infusion rates during the last 30 min of the clamp. Vital function (heart rate, blood pressure, and body temperature) was monitored every 30 min. Only endotoxin administration resulted in mild flu-like symptoms Side effects of LPS occurred in three study participants and consisted of mild flu-like symptoms (mild increase in body temperature up to 38°C, shivering, and feeling cold) with a maximum after 3 h, while other interventions had no side effects

### Heart rate variability (HRV)

R-R intervals were recorded during each intervention over 6 h and during the clamp using a digital SpiderView Holter recorder with seven electrodes to record three-channel ECGs (Sorin Group, Munich, Germany). HRV was analyzed from the Holter monitor recordings using commercially available software (SyneScope version 3.00 analysis system, Sorin Group, Munich, Germany). The sampling rate of the ECG signal was 200 Hz (5 ms resolution). The system automatically edits all artefacts and ectopic beats, and obtains a regular signal by linear interpolation of the heart rate tachogram. Time-domain and frequency-domain parameters of HRV were computed according to the Task Force of the European Society of Cardiology and the North American Society of Pacing and Electrophysiology [7]. Time-domain parameters included mean R-R interval (NN), root mean squared of successive differences (RMSSD), and pNN50 (percentage difference between two consecutive NN intervals over 50 ms). Frequency-domain indices included the LF band (0.04–0.15 Hz), HF band (0.15–0.4 Hz), and LF/HF ratio.

### Indirect calorimetry

Indirect calorimetry was performed in the canopy mode using Vmax Encore 29n (CareFusion, Höchberg, Germany) at baseline, end of intervention, and steady-state clamp conditions for 20 min after 10-min adaptation. Oxygen consumption (VO_2_) and carbon dioxide production (VCO_2_) were measured and respiratory quotient and resting energy expenditure (REE) calculated as previously described [30]. Nonoxidative glucose disposal was calculated from the difference between rate of glucose disappearance and carbohydrate oxidation.

### Metabolites and hormones

Blood samples were immediately chilled, centrifuged, and supernatants stored at -20°C until analysis. Venous blood glucose concentration, serum triglycerides, liver enzymes, cholesterol, fatty acids, C-peptide, insulin, plasma glucagon, cortisol, and GLP-1 were measured as previously described [30). Serum inflammatory markers were assayed using the Quantikine HS (tumor necrosis factor-α [TNF-α], interleukin-6 [IL-6]) and Quantikine (interleukin-1 receptor antagonist [IL-1ra]) ELISA kits (R&D Systems, Wiesbaden, Germany) as previously reported [31].

For gene expression analyses, total RNA was isolated from peripheral blood collected in PAXgene Blood RNA tubes (PreAnalytiX, Hombrechtikon, Switzerland) according to the protocol of the miRNeasy Mini Kit (QIAGEN, Hilden, Germany) including the on-column DNase digestion. RNA quantity and quality were determined by Nanodrop (Peqlab, Erlangen, Germany) and RNA 6000 Nano Kit (Agilent, Böblingen, Germany), respectively. For reverse transcription we used the miScript Reverse Transcription Kit (QIAGEN) for RT-PCR, the QuantiTect SYBR Green PCR Kit and the QuantiTect Primer Assay (QIAGEN). For the relative quantification (RQ) of the gene expression, we used the comparative CT (threshold cycle) method using a threshold of 0.4 and set the baseline between cycles 3 and 15.

### Statistical analysis

Continuous data are expressed as mean±SEM. Non-parametric tests (Spearman rank correlation, Wilcoxon matched-pairs signed rank test, and Friedman test) were applied for data analyses. Incremental area under the curve (AUC) was calculated using the trapezoid method. Univariate linear regression analyses were performed to determine possible correlations between two variables. To determine whether changes of cytokine and incretin concentrations correlate with changes of HRV parameters the following approach was applied. First, deltas (Δ) for each parameter were calculated (Δ1 = 1h–0h, Δ2 = 2h–1h, Δ3 = 3h–2h, Δ4 = 4h–3h). Second, in order to be able to correlate all 64 points (4x16), data were centered (c) towards zero (e.g. cHRΔ1,i = HRΔ1,$\text{i}-\bar{\text{x}}\text{HR}\Delta 1$; cIL1-raΔ1,i = IL1-raΔ1,$\text{i}-\bar{\text{x}}\text{IL}1-\text{ra}\Delta 1$; i = 1 to 16). Analysis of covariance (ANCOVA) was used to compare the slopes of two regression lines. All analyses were performed using IBM SPSS version 22 (IBM, Armonk, NY, USA) and GraphPad Prism version 6.04 statistical software (GraphPad, La Jolla, CA, USA). All tests were two-tailed and the level of significance was set at α = 0.05.

## Results

The main demographic and clinical characteristics of the subjects studied are listed in Table 1 (for further details see ref. [30]). Plasma triglycerides rose continuously from 0.9±0.1 mmol/L at baseline to 3.7±0.7 mmol/L (AUC P<0.01 vs. glycerol) during i.v. fat infusion and remained unchanged after oral fat or LPS. Plasma FFA increased after i.v. fat by 178% from 0.45±0.05 mmol/L at baseline to 0.79±0.04 mmol/L at 360 min (AUC P<0.01 vs. glycerol), but not after oral fat. Plasma FFA started to increase 90 min following endotoxin application to 0.84±0.04 mmol/L (AUC P<0.01 vs. glycerol). Fat infusion, oral fat load, and endotoxin similarly diminished whole-body insulin sensitivity (M-value) to a similar degree to 60, 67, and 48%, respectively (all P<0.01 vs glycerol) (for details see ref. [30]). No correlation was noted between any of the measures of autonomic tone and M-value.

**Table 1:** Demographic and clinical characteristics of the subjects studied.

*n* (male/female)	16 (11/5)
Age (years)	24.4 ± 0.58
BMI (kg/m²)	22.7 ± 0.29
Lean body weight (kg)	58.9 ± 2.09
Body fat (%)	20.6 ± 1.26
Systolic blood pressure (mmHg)*	111 ± 2.05
Diastolic blood pressure (mmHg)*	64.4 ± 1.29
Triglycerides (mmol/L)	0.93 ± 0.08
Fasting blood glucose (mmol/L)	4.37 ± 0.06
2-h blood glucose (mmol/L)	4.43 ± 0.22
Heart rate (bpm)*	62.5 ± 1.99
pNN50 (%)*	27.7 ± 4.18
RMSSD (ms)*	57.1 ± 6.25
Low-frequency power (ms²)*	2012 ± 269
High-frequency power (ms²)*	844 ± 139
LF/HF ratio*	2.79 ± 0.28

* Values at baseline prior to endotoxin infusion.

### LPS

**Fig 1:**
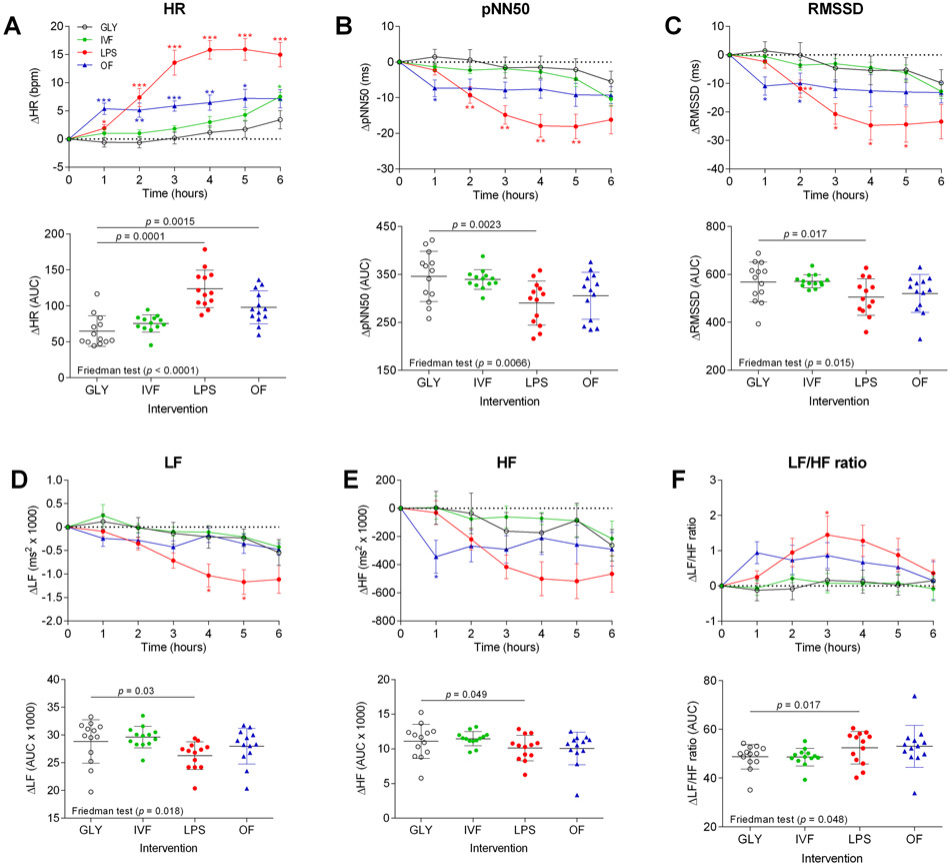
Changes in heart rate and heart rate variability (HRV) indices over 6 hours with the corresponding AUCs during the four interventions studied.

The changes in heart rate and the five HRV measures with the individual AUCs are shown in Fig 1. Compared to glycerol infusion, LPS infusion resulted in increased heart rate at hours 1–6, reduced pNN50 and RMSSD at hours 2–5, diminished LF power at hours 4–5, and increased LH/HF ratio at 3 hours, while the changes between the groups in HF power did not reach statistical significance. In addition, heart rate and LH/HF ratio (AUCs) were significantly larger, whilst pNN50, RMSSD, LF power, and HF power (AUCs) were significantly smaller after LPS exposure.

**Fig 2:**
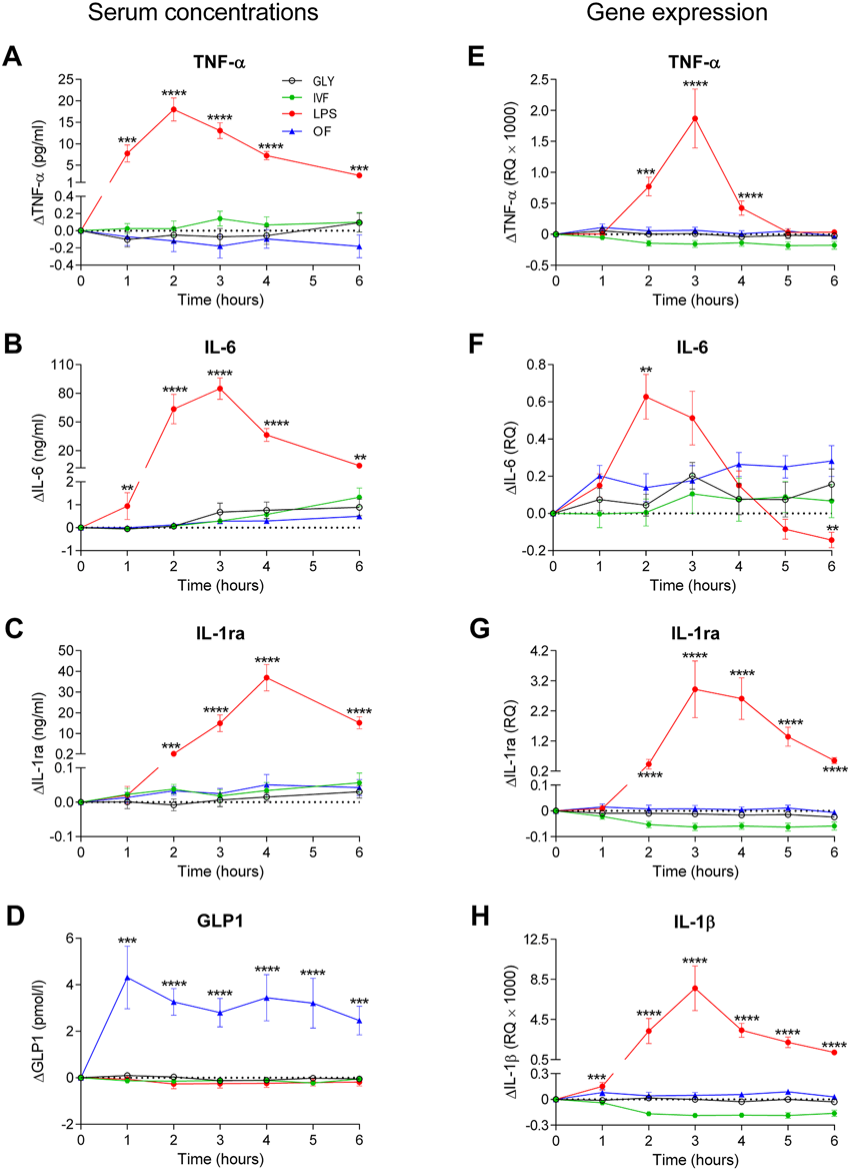
The time course of serum concentrations (A-C) and gene expression (E-H) of pro- and anti-inflammatory cytokines and GLP-1 concentrations (D).

The changes of serum concentrations and gene expression of pro- and anti-inflammatory cytokines and GLP-1 over 6 hours are shown in Fig 2. Following LPS administration, TNF-α and IL-6 concentrations and IL-1ß gene expression increased significantly from baseline to 1–6 hours. After LPS infusion compared with glycerol infusion, IL-1ra concentrations and gene expression rose significantly after 2–6 hours, TNF-α gene expression was significantly elevated after 2–4 hours compared to baseline, while IL-6 gene expression was increased after 2 hours only.

### Oral fat

Following oral fat intake, mean heart rate rose significantly at hours 1–5 and heart rate AUC was significantly larger compared to glycerol infusion. After oral fat intake, pNN50 and HF power were significantly reduced at 1 hour, while RMSSD was significantly diminished after 1–2 hours compared to glycerol infusion (Fig 1). GLP-1 concentrations after oral fat intake were significantly elevated as compared with glycerol infusion after 1–6 hours (Fig 2).

### Intravenous fat

After i.v. fat, heart rate was significantly increased at 6 hours compared to glycerol infusion. No other significant changes in comparison with glycerol were observed.

### Correlation analyses

The correlations between the changes in heart rate and HRV indices and changes in IL-1ra concentrations and IL-1ra and IL-1ß gene expression following LPS infusion are shown in Table 2. The changes in IL-1ra levels and IL-1ra and IL-1ß gene expression showed an inverse correlation with the changes in RMSSD, LF power, and HF power and a positive correlation with LF/HF ratio (all P<0.05), except for three associations showing borderline significance (ΔIL-1ra gene expression vs ΔHF: P = 0.051, ΔIL-1ß gene expression vs ΔLF: P = 0.087, and ΔIL-1ß gene expression vs ΔHF: P = 0.078). The changes in heart rate and pNN50 were significantly associated with those of IL-1ra concentrations (both p<0.05), but not with the changes in IL-1ra and IL-1ß gene expression. No other meaningful correlations were found.

**Table 2:** Correlation coefficients (Spearman) for the relations between the changes in heart rate and HRV indices and changes in IL-1ra concentrations and IL-1ra and IL-1ß gene expression during endotoxemia.

	ΔIL-1ra (ng/ml)		ΔIL-1ra (RQ)		ΔIL-1β (RQ)	
	r	*P*	r	*P*	r	*P*
**ΔHeart rate (bpm)**	0.268	0.039	0.067	0.646	0.179	0.219
**ΔpNN50 (%)**	-0.287	0.026	-0.024	0.877	-0.147	0.330
**ΔRMSSD (ms)**	-0.361	0.0046	-0.335	0.0019	-0.237	0.03
**ΔLF (ms** ^**2**^ **)**	-0.417	0.0009	-0.323	0.0027	-0.188	0.087
**ΔHF (ms** ^**2**^ **)**	-0.288	0.026	-0.214	0.051	-0.194	0.078
**ΔLF/HF ratio**	0.341	0.0076	0.353	0.001	0.395	0.0002

**Fig 3:**
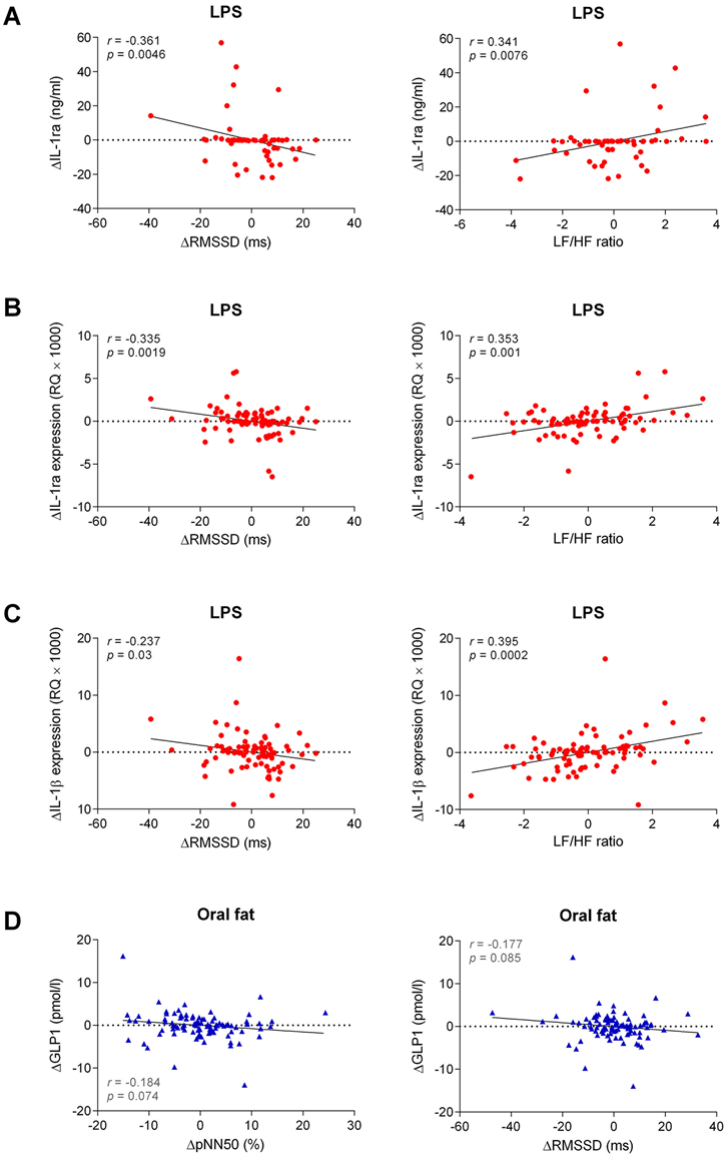
Correlations of the changes in IL-1ra serum concentrations (A), IL-1ra expression (B), and IL-1ß expression (C) with RMSSD and low-frequency/high-frequency (LF/HF) ratio following LPS infusion and correlations of the changes in GLP-1 with pNN50 and RMSSD after oral fat intake (D).

Fig 3 illustrates the correlations of the changes in IL-1ra serum concentrations, IL-1ra expression, and IL-1ß expression with RMSSD and LF/HF ratio following LPS infusion and correlations of the changes in GLP-1 with pNN50 and RMSSD after oral fat intake. The changes in GLP-1 concentrations following oral fat intake tended to be inversely correlated with pNN50 (P = 0.074) and RMSSD (P = 0.085), but not with the frequency domain measures.

The comparisons of the regression slopes between LPS and glycerol interventions resulting from the correlations of heart rate and HRV indices (AUCs) with lipid and glucose oxidation after 6 hours and M-value are shown in Table 3. The regression slopes of heart rate, pNN50, and RMSSD (AUCs) with lipid oxidation and glucose oxidation (vice versa) were significantly different between LPS and glycerol interventions. A trend toward a difference between LPS and glycerol interventions was observed for the regression slopes of HF power (AUC) with lipid oxidation (P = 0.06) and glucose oxidation (P = 0.057), whereas the regression slopes for LF power and LH/HF ratio (AUCs) were not different. No correlations of heart rate and HRV indices AUCs with insulin sensitivity were observed.

**Table 3:** Comparison of the regression slopes between LPS and glycerol interventions resulting from the correlations of heart rate and HRV indices (AUCs) with lipid oxidation, glucose oxidation, and M-value.

	Lipid oxidation (n = 14)		Glucose oxidation (n = 14)		M-value (n = 15)	
	*β*	*P* value	*β*	*P* value	*β*	*P* value
Heart rate AUC	0.732	0.003	-0.526	0.006	-0.047	0.876
pNN50 AUC	-0.922	0.014	0.729	0.009	0.115	0.783
RMSSD AUC	-0.888	0.033	0.706	0.023	0.425	0.343
LF power AUC	-0.360	0.397	0.456	0.151	-0.604	0.173
HF power AUC	-0.842	0.060	0.638	0.057	0.445	0.346
LF/HF ratio AUC	0.524	0.230	-0.257	0.439	-0.277	0.552

## Discussion

The results of this study demonstrate that the suppression of cardiac vagal activity and sympathetic predominance during endotoxemia are predominantly linked to the anti-inflammatory IL-1ra response to IL-1ß activation rather than to the pro-inflammatory response by IL-6 or TNF-α. The reduction in vagal but not sympathetic tone was associated with increased lipid oxidation and diminished glucose oxidation. The effect of oral high-fat load on autonomic tone showed a different pattern with a faster onset, but considerably weaker increase in heart rate and only modest selective decline in time domain HRV indicating slightly suppressed vagal tone which tended to be associated with the post-load GLP-1 response. In contrast, i.v. lipid infusion did not influence the cardiac autonomic tone. The observed changes in autonomic tone were not related to insulin sensitivity assessed by the M-value obtained from the subsequent hyperinsulinemic-euglycemic clamp.

One major novel finding is the link between the suppression of cardiac vagal tone during endotoxemia and the anti-inflammatory response. This contrasts with previous studies in healthy humans which failed to show a relationship between the IL-1ra response to LPS and HRV changes [17] or reported a positive correlation of baseline vagal activity with the maximal LPS-induced TNF-α response [16], and with *in vitro* data suggesting that higher vagal tone during paced breathing was associated with lower LPS-stimulated production of TNF-α and IL-6, but was not related to the production of the anti-inflammatory cytokine IL-10 [32]. Instead, our data is in line with the finding that rhIL-10 administration in humans suppresses not only the LPS-induced inflammatory response but also the heart rate elevation [33]. Our finding in this respect is also compatible with recent experimental work demonstrating that in vagotomized endotoxemic rats, the inflammatory response was aggravated with elevated IL-1ß in both plasma and ventricular tissue accompanied by pronounced cardiac impairment [6], suggesting that the heart plays a pivotal role in the context of the “cholinergic anti-inflammatory reflex” [1,5,6]. Vagus nerve stimulation suppresses local and serum proinflammatory cytokine levels in endotoxemic rodents, and acetylcholine inhibits the release of cytokines including IL‑1β from LPS-stimulated macrophages [2,3], suggesting that the efferent vagus nerve-based arm of the inflammatory reflex represents the cholinergic anti-inflammatory pathway [2]. The proinflammatory cytokine IL-1ß has been proposed as an important mediator of neuroimmune interactions, which also participates directly in neurodegeneration. Activation of primary mixed glial cultures by incubation with LPS caused marked increases in release of IL-6 and nitric oxide which were inhibited by co-incubation with IL-1ra [34]. In cultured myenteric plexus cells, LPS altered the interaction between myenteric neurons and enteric glial cells via stimulating IL-1ß secretion from enteric glial cells, and these effects were blocked by pretreatment with IL-1ra [35].

The correlation between LPS-induced reduction in cardiac vagal tone and augmented fat oxidation observed herein deserves a brief comment. The majority of human myocardial energy supply in the resting state comes from fat oxidation which increases with greater heart work during atrial pacing, with a similar relative proportion of fat oxidation to total myocardial energy expenditure [36]. The ANS plays an important role in controlling the selection of fuel by the heart, since the rate of myocardial glucose oxidation is markedly impaired in the chronically denervated heart [37], likely due to a decline in myocardial pyruvate dehydrogenase [38]. It has been suggested that the decline in glucose oxidation is compensated by an enhanced contribution of fatty acid oxidation to overall oxidative energy conversion [39]. Our findings are in line with these concepts and with recent data indicating that experimental vagal stimulation reduces FFA oxidation during mild ß-adrenergic stress [40] and that human endotoxemia increases lipid oxidation [41].

Another novel finding observed herein is the augmented heart rate and suppressed autonomic tone, albeit to a slight degree, in relation to the GLP-1 response following oral high-fat loading. In healthy individuals during hyperglycemia, GLP-1 infusion led to an average increase in heart rate by 3 bpm and to a slight, statistically non-significant decrease in cardiac vagal tone [42]. It has been argued that this does not exclude the possibility that GLP-1-induced vagal depression could have been greater in the absence of prior hyperglycemia and hyperinsulinemia [29]. This notion is supported by experimental data showing that central GLP-1 receptor stimulation increased heart rate and diminished cardiac parasympathetic activity by inhibiting neurotransmission to cardiac vagal neurons [43]. In healthy humans, low-dose GLP-1 infusion reaching physiological plasma levels of GLP-1 corresponding to peak levels found after oral ingestion of a high caloric glucose meal reversibly diminished pancreatic polypeptide (PP), a marker of parasympathetic outflow to the gut, with and without administration of duodenal lipid, indicating inhibition of efferent vagal-cholinergic activity [28]. Against this background, it is conceivable that GLP-1 release may have contributed to the slight suppression of vagal tone after oral fat load observed herein.

The strength of the present work is the comprehensive array of interventions and metabolic, immune, hormonal, and autonomic function assessments allowing us to address distinct questions. Study limitations that could be potential sources of bias include first, the relatively small sample size, particularly given that the effects of oral fat on HRV and its correlation with GLP-1 were borderline and in view of the relatively high intraindividual variability of the frequency domain HRV measures. It is possible that a larger sample size could result in a more precise effect estimate of this intervention. Second, we used a low-dose (0.5 ng/kg body weight) LPS model, whereas the majority of published studies used higher LPS doses (2–4 ng/kg body weight). It is well known that cytokine dynamics after endotoxin are dose dependent [20]. Higher doses of E. coli LPS, such as 2–4 ng/kg body weight, cause an almost supraphysiological (up to 2000-fold) increase in TNF-α [20]. However, it has been suggested that doses of 0.3–1 ng/kg body weight which result in a 3–100 fold rise in TNF-α resemble the concentrations during human sepsis more accurately [20]. Third, plasma catecholamines were not determined which could have contributed to elucidate a possible relationship between LPS-induced SNS activation and the cytokine response. Epinephrine infusion has been shown to attenuate pro-inflammatory cytokine levels [15,44] and facilitates the anti-inflammatory response during human endotoxemia [44], but further reduces vagal activity [15]. However, whether LPS-induced rise in catecholamines [19] is linked to the increase in anti-inflammatory cytokine levels has not been reported.

We are confident that our data open up a potential therapeutic perspective, since vagus nerve signaling has an important role in the regulation of feeding behavior and metabolic homeostasis and activation of cholinergic signaling in the efferent arm of the inflammatory reflex alleviates obesity-associated inflammation and metabolic derangements [2]. This study conducted in healthy volunteers shows that an experimental condition mimicking inflammation leads to suppression of cardiac vagal tone and sympathetic predominance which are linked to the anti-inflammatory response and to increased lipid oxidation, while oral high-fat load results in a modest decline in vagal tone in relation to the postprandial incretin response. Vagus nerve activities regulating hepatic glucose metabolism and cardiac function are both impaired in obesity suggesting that augmentation of efferent vagus nerve activity, e.g. by cholinergic agents or devices for vagus nerve stimulation and anti-inflammatory modulators or biofeedback to treat disease of cytokine excess could be beneficial in patients with hyperglycemia, metabolic syndrome, and cardiovascular disease [2,45].

In conclusion, we showed that 1.) suppression of cardiac vagal tone and sympathetic predominance during endotoxemia are linked to the anti-inflammatory response and to increased lipid oxidation and diminished glucose oxidation, 2.) oral high-fat load but not lipid infusion resulted in a slight increase in heart rate and decline in vagal tone in relation to postprandial GLP-1 response, and 3.) the changes in autonomic tone were not related to subsequent reduction in insulin sensitivity.
